# Prevalence of Adverse Effects Associated With Transcranial Magnetic Stimulation for Autism Spectrum Disorder: A Systematic Review and Meta-Analysis

**DOI:** 10.3389/fpsyt.2022.875591

**Published:** 2022-05-23

**Authors:** Zhang Huashuang, Li Yang, Hou Chensheng, Xin Jing, Chen Bo, Zhang Dongming, Liang Kangfu, Wang Shi-Bin

**Affiliations:** ^1^Institute for Brain Research and Rehabilitation, South China Normal University, Guangzhou, China; ^2^Department of Ophthalmology, Affiliated Foshan Hospital, Southern Medical University, Foshan, China; ^3^Center for Evidence-Based and Translational Medicine, Zhongnan Hospital of Wuhan University, Wuhan, China; ^4^Department of Pediatric Rehabilitation Medicine, Foshan Fosun Chancheng Hospital, Foshan, China; ^5^Department of Cardiovascular Surgery, The People's Hospital of Gaozhou, Gaozhou, China; ^6^Department of Neurology, Foshan Fosun Chancheng Hospital, Foshan, China; ^7^Guangdong Mental Health Center, Guangdong Provincial People's Hospital, Guangdong Academy of Medical Sciences, Guangzhou, China

**Keywords:** autism spectrum disorder, transcranial magnetic stimulation, adverse effects, systematic review, meta-analysis

## Abstract

**Background:**

A growing number of studies have suggested that transcranial magnetic stimulation (TMS) may represent a novel technique with both investigative and therapeutic potential for autism spectrum disorder (ASD). However, a full spectrum of the adverse effects (AEs) of TMS used in ASD has not been specifically and systematically evaluated.

**Objective:**

This systematic review and meta-analysis was to assess the prevalence of AEs related to TMS in ASD and to further explore the potentially related factors on the AEs.

**Methods:**

A systematic literature research of articles published before 31 December 2020 was conducted in the databases of PubMed, Embase, Cochrane Library, Ovid, PsycINFO, Chinese National Knowledge Infrastructure (CNKI), Chongqing VIP, and WANFANG DATA. AEs reported in the studies were carefully examined and synthesized to understand the safety and tolerability of TMS among ASD. Then, subgroup and sensitivity analyses were performed to examine the potentially related factors on the AEs. PROSPERO registration number: CRD42021239827.

**Results:**

Eleven studies were included in the meta-analysis. The pooled prevalence with 95% confidence interval (CI) of AEs was calculated (overall AEs: 25%, 95% CI 18–33%; headache: 10%, 95% CI 3–19%; facial discomfort: 15%, 95% CI 4–29%; irritability 21%, 95% CI 8–37%; pain at the application site: 6%, 95% CI 0–19%; headedness or dizziness: 8%, 95% CI 0–23%). All reported AEs were mild and transient with relatively few serious AEs and can be resolved after having a rest or medication. In addition, the following variables showed no significant change in overall prevalence of AEs: the purpose of using TMS, mean age of participants, whether the stimulation site was dorsolateral pre-frontal cortex (DLPFC), intensity of TMS, and the number of stimulation sessions.

**Conclusion:**

The overall prevalence of reported AEs of TMS among ASD was 25%. No identified ASD-specific risk factors for TMS-induced AEs were found. Further studies are needed to clarify the variation in the prevalence.

**Systematic Review Registration:**

www.crd.york.ac.uk/PROSPERO/display_record.php?RecordID=239827, PROSPERO, identifier: CRD42021239827.

## Introduction

Autism spectrum disorder (ASD) refers to a group of complex neurodevelopmental disorders characterized by impaired communications, restricted and repetitive behaviors, and limited social interactions (1). In 2010, there were estimated 52 million cases of ASD, equating to a prevalence of 7.6 per 1,000 or 1 in 132 persons (2). For children aged from 5 to 14 years, ASD acted as the 4th factor primarily causing disability out of the mental disorders (2). Staggering reality lies on the clinical, social, and financial burden of ASD. We need valid and trustworthy biomarkers for diagnosis and effective treatments targeting ASD (3).

Over the past years, the availability of transcranial magnetic stimulation (TMS), a non-invasive brain stimulation technique, has given the hope that it could be one effective tool for treating ASD (4). TMS refers to an approach to achieve a non-invasive focal brain stimulation process, in which localized intracranial electrical currents, sufficiently significant for depolarizing a faction of neurons, received the generation from fast varying extracranial magnetic fields (5). Thus, far, an increasing number of studies have reported the efficiency of high- and low-frequency TMS on ASD. It is promising for TMS to treat ASD (6–11). In addition, TMS may have experimental prospects among ASD, because the development of novel treatment for such complex and heterogeneous disorders, such as ASD, requires a deeper understanding of the underlying pathophysiology (3). TMS was used as an experimental tool to understand the pathophysiology of ASD by several research teams (12–16). Therefore, TMS can be used both experimentally and therapeutically among ASD (3).

To date, TMS is considered relatively safe, even in the pediatric population (17, 18). However, studies have showed that TMS does pose some risk for adverse effects (AEs) (19). Even though the study by Rossi et al. (20) has updated the previous safety guidelines from 2009, there are no studies specifically targeting at ASD population. Thereby, it is vitally important to implement a systematical and broad assessment on the AEs of the stimulation previous to large-scale promotion of TMS among ASD. Nevertheless, when it comes to the AEs, the related studies of assessing TMS in the ASD population are sparse. Although a previous study (21) has reported the AEs related to TMS, it was not specifically targeted at the ASD population. It is universally known that ASD is a highly heterogeneous group of patients. The results mixed with other kinds of patients' data are not necessarily suitable for further understanding of ASD. In addition, there are no established and specialized guidelines for using TMS in ASD. TMS is recommended by the current safety guidelines with caution (3, 19). As TMS in the ASD Consensus Group indicated, a gap in the preliminary studies of TMS in ASD (as well as other conditions) is the lack of a systematic effort to identify, track, and report AEs in study publications (22). Thus, we performed a systematic review and meta-analysis to assess the prevalence of AEs related to TMS used both as a therapeutic intervention and an experimental tool in ASD. Then, we further explored the potentially related factors on the AEs.

## Methods

### Protocol

The protocol of the current systematic review was registered on the International Prospective Register of Systematic Reviews (PROSPERO; registration number: CRD42021239827). Our systematic review was conducted according to the recommendations of the Cochrane AEs Methods Group (23). In addition, this systematic review used Preferred Reporting Items for Systematic Reviews and Meta-Analyses (PRISMA) guidelines for conducting and reporting systematic reviews (24).

### Study Selection: Inclusion and Exclusion Criteria

Studies were included if they met the following criteria: (1) participants: diagnosed with ASD according to the following criteria: Diagnostic and Statistical Manual of Mental Disorders, Fourth Edition (DSM-IV) or Diagnostic and Statistical Manual of Mental Disorders, Fifth Edition (DSM-5) or Autism Diagnostic Observation Schedule-2 (ADOS-2) or International Classification of Diseases, tenth revision (ICD-10); (2) intervention: original articles used TMS either as an intervention or as an investigative tool without any other stimulation (e.g., transcranial direct current stimulation); (3) outcome: the AEs related to TMS used in ASD were reported in the papers; and (4) study design: randomized controlled design or case–control design or crossover design or open-label, single-arm design, or case series. For randomized controlled design and case–control design, we only extracted the data of group that received TMS and excluded the data from the control group. We excluded the following articles: (1) articles that included patients with brain damage, such as tumors, (2) meta-analysis on reporting duplicate data or data extracted from original articles, (3) animal studies, (4) review articles, and (5) abstract or case report.

### Search Strategy

Articles published before 31 December 2020 were searched in the following databases: PubMed, Embase, Cochrane Library, Ovid, PsycINFO, Chinese National Knowledge Infrastructure (CNKI), Chongqing VIP, and WANFANG DATA. The following keywords and search strategy in either Chinese or English were used: (((((((((transcranial magnetic stimulations[Title/Abstract]) OR (tms[Title/Abstract])) OR (transcranial magnetic stimulation[Title/Abstract])) OR (single pulse transcranial magnetic stimulation[Title/Abstract])) OR (paired pulse transcranial magnetic stimulation[Title/Abstract])) OR (repetitive transcranial magnetic stimulation[Title/Abstract])) OR (theta burst stimulation[Title/Abstract])) OR (tbs[Title/Abstract])) OR (“Transcranial Magnetic Stimulation”[Mesh])) AND (((((autism spectrum disorder[Title/Abstract]) OR (autism spectrum disorders[Title/Abstract])) OR (Asperger[Title/Abstract])) OR (pervasive developmental disorder[Title/Abstract])) OR (“Autism Spectrum Disorder”[Mesh])).

### Data Extraction

For each study, data were extracted independently by two authors (CB & ZD). The distinction, each, can be tackled by conformity to another author (ZH) as a consultant under necessary conditions. Our group did deliver elaboration on a structured checklist, aiming to contribute extracted variables as follows:

The primary outcome measures: AEs related to TMS.

In addition, we also extracted the following demographic characteristics and TMS methodology: diagnosis criteria, sample size, gender, mean age and mean IQ in both the active group and sham/control group, types of TMS, brain regions of stimulation, motor threshold (MT), frequency, total stimulation, and the number of stimulation sessions and methods of measuring AEs.

### Risk of Bias Assessment

Three independent authors performed the quality assessment of the articles included in the systematic review using the Newcastle-Ottawa Scale (NOS) (25). The scores of the NOS range between 0 and 9. The score of a study is classified into one of the three groups based on the score, namely, low (≤ 4), moderate (between 5 and 6), and high-quality (≥7) study.

### Statistical Analysis

Meta-analysis was performed using the R software (version 4.0.3) where the prevalence of the AEs was pooled using the inverse variance method. For heterogeneity between studies was not high (*I*^2^ <50% and *p* > 0.1), a common effect model was adopted for the calculation of AEs rate; otherwise, a random-effects model will be used. Publication bias was examined by visual inspection of the funnel plot and tested by Egger's test and Begg's test. To identify moderators or mediators of the effect on the prevalence of AEs, five subgroups and sensitivity analyses were conducted by contrasting the following factors: (1) clinical studies vs. basic studies; (2) studies with participants' mean age >18 years vs. those with participants' mean age ≤ 18 years; (3) studies with stimulation targeted at dorsolateral pre-frontal cortex (DLPFC) vs. those without stimulation targeted at DLPFC; (4) studies using MT ≤ 90% vs. those using MT >90%; and (5) number of stimulation sessions <10 vs. number of stimulation sessions ≥10. All statistical tests were two-sided with α = 0.05, and *p* < 0.05 suggesting statistical significance.

## Results

### Study Selection

**Figure 1:**
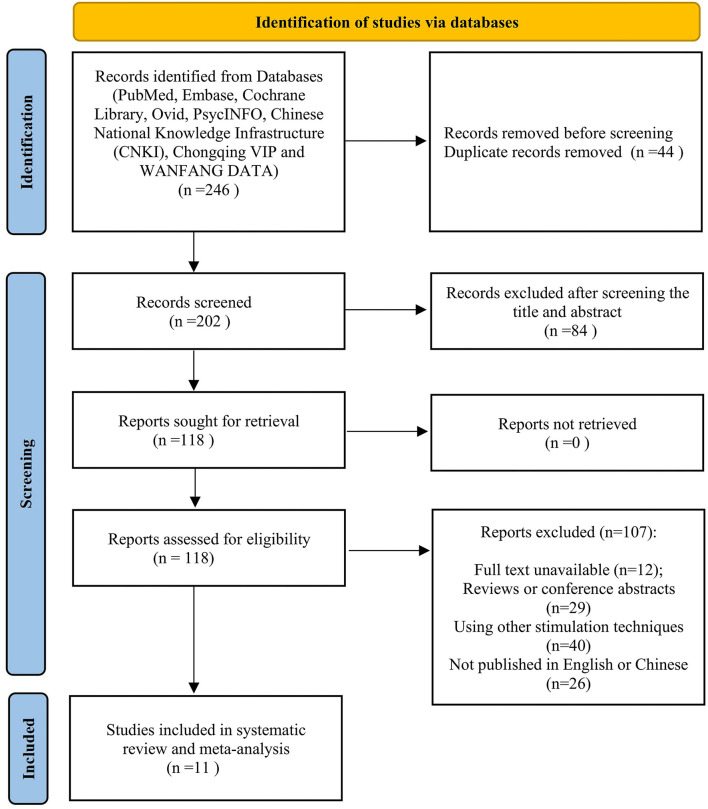
PRISMA 2020 flowchart selection of studies.

Overall, 246 papers were collected and 202 records remained after duplicates were removed. Moreover, 84 papers were excluded after screening on titles and abstracts due to the irrelevant topic. Then, the rest of the papers were screened by means of the full-text review. Among the remained 118 studies, 107 papers were removed because they were conference abstracts and reviews, based on other stimulation techniques or with no full-text available. Finally, adequate data on AEs can be found in 11 papers for later performing meta-analysis (Figure 1).

### Study Characteristics

A detailed description of the included studies is shown in Tables 1, 2. All studies recruited subjects diagnosed with ASD as defined by DSM-IV, DSM-5, or ADOS-2. Age caused a difference in the participants from the enrolled studies, among which some targeted children/adolescents and others only enrolled adults. The mean age of participants ranged from 7.09 to 36.60 years. According to the mean IQ shown in Table 1, we can find that all included participants in the active groups were high-functioning ASD or Asperger's disorder but those in the included study by Yang et al. (28). Thus, the assessment of AEs for these participants mainly depended on questionnaire or interview. However, for the low-functioning autism in the included study of Yang et al. (28), the assessment of AEs was obtained by asking the caregivers before and after each TMS session. Such assessment method was mainly due to the communication difficulties of low-functioning autism. The neurostimulation parameters among studies varied considerably. The frequencies employed ranged from 1 Hz to 50 Hz, with 2 articles using 1 Hz (13, 27), one article using 5 Hz (6), 5 articles using ≤ 30 Hz (10, 14, 28, 29, 31), and 3 articles using 50 Hz (15, 26, 30). The intensity was expressed as MT% from 50% to 120%, with 8 articles using ≤ 90% (10, 13–15, 26–28, 30) and 3 articles using >90% (6, 29, 31). As for brain regions of stimulation, Ni et al. (26) involved in two regions, including DLPFC and posterior superior temporal sulcus (pSTS); 5 studies (6, 10, 27, 29, 30) stimulated the DLPFC; the other 5 studies (13–15, 28, 30) did not target at DLPFC. Among the enrolled 11 papers in meta-analysis, one was case series (28), 3 studies lacked a sham control group (28, 29, 31), and 2 studies were randomized crossover design (13, 26). Randomization and blinding were only considered in 5 studies (6, 10, 14, 15, 27). All the included studies were reported in English except for one study (31) written in Chinese. In addition, there was an interval of 5 and 7 days between two sessions of TMS in the crossover designs by Fecteau et al. (13) and Ni et al. (26), respectively. Such existence of interval was used to remove the residual effects. Several studies have identified that the effect of one session of TMS did not last for 1 week (32–34). Therefore, the data from these crossover studies could be used for meta-analysis. In addition, all included studies checked the history of epilepsy during the process of recruiting participants and did not include any individuals with comorbid epilepsy.

**Table 1:** Description of selected studies in meta-analysis.

**No**.	**Study**	**Diagnosis** **(Criteria)**	**Sample size**		**Gender**		**Mean age**		**Mean IQ**		**Assessment of AEs**	**NOS**
			**Active group**	**Sham/** **control group**	**Active group**	**Sham/** **control group**	**Active group**	**Sham/** **control group**	**Active group**	**Sham/** **control group**		
1	Pedapati et al. (14)	ADOS-2	9	9	7/2	5/4	15.60	14.5	106.20	n/a	Review	6
2	Ameis et al. (10)	DSM-IV ADOS-2	20	20	14/6	14/6	23.50	21.65	100.90	100.45	Interview	8
3	Fecteau et al. (13)	DSM-IV	10	10	7/3	7/3	36.60	36.60	122.40	111.20	Questionnaire	7
4	Enticott et al. (6)	DSM-IV	15	13	13/2	10/3	33.87	30.54	n/a	n/a	n/a	7
5	Jannati et al. (15)	DSM-5, ADOS-2	11	18	n/a	n/a	13.09	13.44	103.55	n/a	n/a	4
6	Ni et al. (26)	DSM-IV, ICD-10, ADI-R, ADOS	19	19	14/5	14/5	20.80	20.80	100.5	100.5	n/a	4
7	Baruth et al. (27)	DSM-IV, ADI-R	16	20	n/a	12/8	13.90	15.30	86.0	n/a	Review	5
8	Yang et al. (28)	DSM-5, ADI-R, ABC	11	n/a	7/4	n/a	7.09	n/a	<70	n/a	Reported by caregivers	6
9	Gwynette et al. (29)	DSM-5	10	n/a	9/1	n/a	25.50	n/a	>60	n/a	n/a	4
10	Oberman et al. (30)	DSM-IV, Asperger's Syndrome, PDD-NOS, ADOS	19	n/a	19/0	n/a	12.26	n/a	100.42	n/a	n/a	4
11	Dang et al. (31)	DSM-IV	12	n/a	11/1	n/a	7.10	n/a	n/a	n/a	n/a	5

*AEs, Adverse effects; ADOS-2:Autism Diagnostic Observation Schedule, Second Edition; DSM-IV, Diagnostic and Statistical Manual of Mental Disorders, Fourth Edition; DSM-5: Diagnostic and Statistical Manual of Mental Disorders, Fifth Edition; ICD-10, International Classification of Diseases, Tenth Revision; ADI-R, Autism Diagnostic Interview, Revised; ABC, aberrant behavior checklist; PDD-NOS: pervasive developmental disorder not otherwise specified; ADOS, Autism Diagnostic Observation Schedule; IQ, intelligence quotient; NOS, Newcastle-Ottawa Scale*.

**Table 2:** Description of TMS parameters in the selected studies in meta-analysis.

**No**.	**Study**	**Type of TMS**	**Brain regions of stimulation**	**MT, %**	**Frequency, hertz**	**Total stimulation**	**Stimulation sessions**
1	Pedapati et al. (14)	iTBS	Motor cortex	70	30	300	1
2	Ameis et al. (10)	rTMS	DLPFC	90	20	1500*20	20
3	Fecteau et al. (13)	rTMS	Pars opercularis, PTr	70	1	n/a	4
4	Enticott et al. (6)	Deep rTMS	Bilateral DLPFC	110	5	1500*10	10
5	Jannati et al. (15)	cTBS	Left M1	80	50	600	1
6	Ni et al. (26)	iTBS	Bilateral DLPFC, bilateral pSTS	80 for active, 60 for sham	50	600*2	10
7	Baruth et al. (27)	rTMS	DLPFC	90	1	150*12	12
8	Yang et al. (28)	rTMS	Left IPL	50	20	n/a	6
9	Gwynette et al. (29)	rTMS	Left DLPFC	120	10	3000*25	25
10	Oberman et al. (30)	cTBS	M1	80	50	600	1
11	Dang et al. (31)	rTMS	Bilateral DLPFC	100	0–30	n/a	32

*AEs, Adverse effects; TM, transcranial magnetic stimulation; MT, motor threshold; iTBS, intermittent theta burst stimulation; M1, motor cortex; rTMS, repetitive transcranial magnetic stimulation; DLPFC, dorsolateral prefrontal cortex; IPL, inferior parietal lobule; PTr, pars triangularis; cTBS, continuous theta-burst stimulation*.

### Risk of Bias Assessment

**Figure 2:**
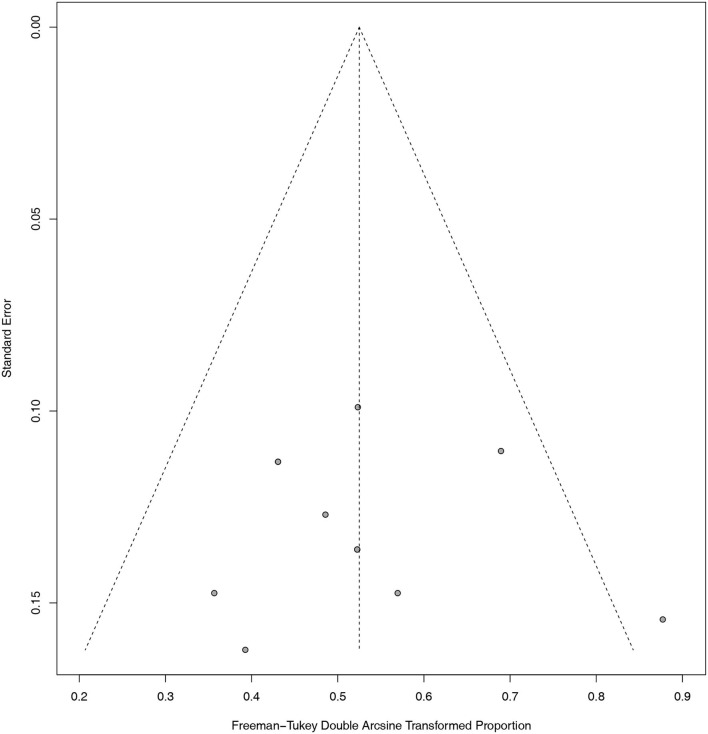
Funnel plot.

The overall quality scores of the NOS scale for the included studies ranged from 4 to 8 (Table 1). The detailed methodological quality of included studies according to the NOS scale was presented in the Supplementary Materials (Supplementary Table 1). In addition, as shown in the funnel plot (Figure 2), we observed that only one study exceeded the limits of the graph. Publication bias was excluded by visualizing the funnel plot of standard error. Egger's test with *p* = 0.8726 and Begg's test with *p* = 0.6354 also suggested that the publication bias was not significant. As for statistical heterogeneity, the values of I^2^ in all meta-analyses were lower than 20%.

### Prevalence of Adverse Effects

**Figure 3:**
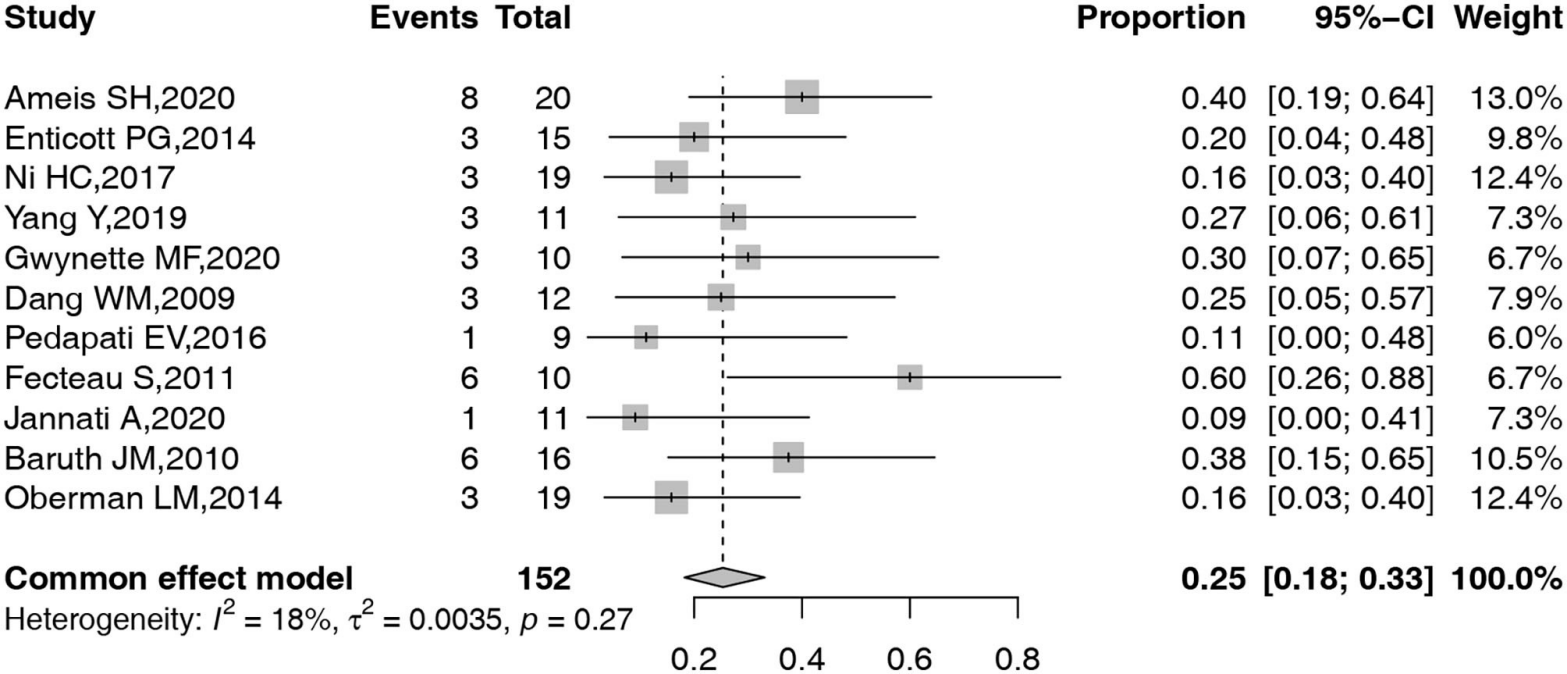
Forest plot of overall pooled prevalence of AEs.

A total of 11 studies, including 155 participants, reported the detailed information on AEs related to TMS in ASD. A common effect model was adopted to obtain the pooled overall prevalence of AEs. The forest plot for the pooled prevalence of these common AEs is shown in Figure 3. Given the low-level heterogeneity, a common effect model was constructed to complete all the above meta-analyses. The overall prevalence of AEs was 25% (95% confidence interval [CI]: 18–33%).

The frequency of all observed AEs is summarized in Table 3, while the number of AEs in each included study is shown in Table 4. As shown in Table 3, the observed AEs included headache, irritability, itching, facial discomfort, sleepy, improved mood, pain at the application site, headedness/dizziness, trouble concentrating, fatigue, stiff neck, mild scalp irritation, neck pain, nausea, more emotional, transient muscle spasms, seizure due to a programming error, subtle disorientation, improved concentration, and other AEs.

**Table 3:** All adverse effects, sorted by frequency.

**Specific adverse effects observed**	**Frequency**	**Proportion**
Headache	8	16%
**Irritability**	**7**	**14%**
Itching	5	10%
Facial discomfort	5	10%
Sleepy	3	6%
Improved mood	3	6%
Pain at application site	2	4%
Headedness/dizziness	2	4%
Trouble concentrating	2	4%
Fatigue	2	4%
Stiff neck	2	4%
Mild scalp irritation	1	2%
Neck pain	1	2%
Nausea	1	2%
More emotional	1	2%
Transient muscle spasms	1	2%
Seizure due to a programming error	1	2%
Subtle disorientation	1	2%
Improved concentration	1	2%
Other	1	2%
In total	50	100%

**Table 4:** Description of the number of adverse effects in each selected study in meta-analysis.

**Detailed type of AEs**	**Pedapati et al. (14)**	**Ameis et al. (10)**	**Fecteau et al. (13)**	**Enticott et al. (6)**	**Jannati et al. (15)**	**Ni et al. (26)**	**Baruth et al. (27)**	**Yang et al. (28)**	**Gwynette et al. (29)**	**Oberman et al. (30)**	**Dang et al. (31)**	**Total**
Total number of participants in active group	9	20	10	15	11	19	16	11	10	19	12	152
Total number of participants that experienced adverse effects	1	8	6	3	1	3	6	3	3	3	3	40
Itching							5					5
Irritability								3	1		3	7
Headache	1	4	1				1			1		8
Sleepy			3									3
Headedness/dizziness			1	1								2
Facial discomfort				2		3						5
Mild scalp irritation					1							1
Pain at application site		1	1									2
Neck pain		1										1
Nausea		1										1
Improved mood			3									3
Trouble concentrating			2									2
More emotional			1									1
Transient muscle spasms									1			1
Seizure due to a programming error									1			1
Fatigue										2		2
Stiff neck			2									2
Subtle disorientation			1									1
Improved concentration			1									1
Other		1										1

**Figure 4:**
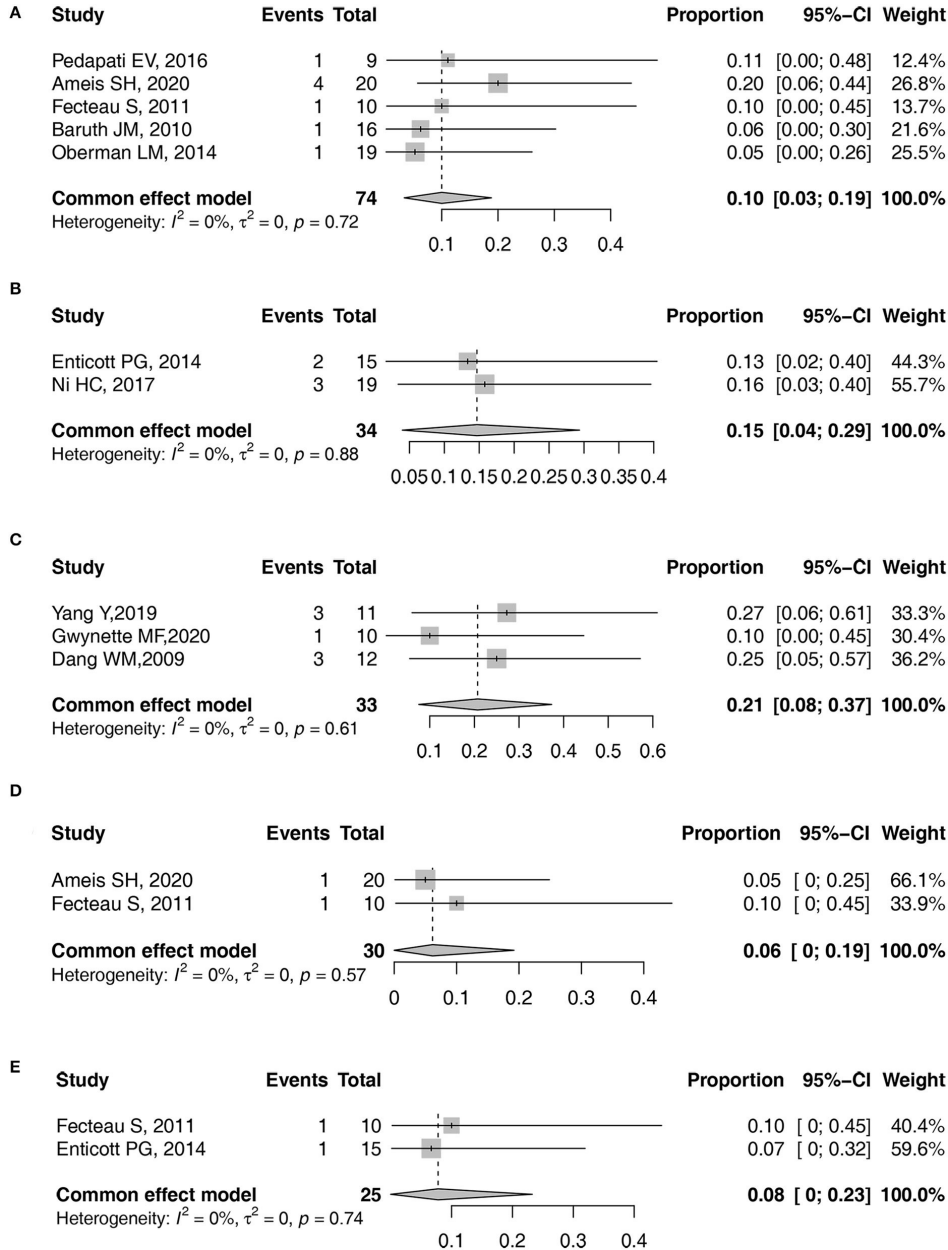
Forest plots of pooled prevalence. **(A)** Headache. **(B)** facial discomfort. **(C)** irritability. **(D)** pain at the application site. **(E)** headedness/dizziness.

The pooled prevalence with 95% CI of headache, facial discomfort, irritability, pain at the application site, and headedness/dizziness was obtained as 10% (95% CI: 3–19%), 15% (95% CI: 4–29%), 21% (95% CI: 8–37%), 6% (95% CI: 0–19%), and 8% (95% CI: 0–23%), respectively (Figure 4). However, due to the low number of studies that reported other AEs, a meta-analysis of other AEs prevalence could not be conducted.

### Subgroup and Sensitivity Analyses

The subgroup analyses showed that no significant differences were found in the prevalence rates of AEs between groups by the following variables: the purpose of using TMS, mean age of participants, whether the stimulation site was DLPFC, intensity of TMS, and the number of stimulation sessions (refer to Table 5).

**Table 5:** Subgroup and sensitivity analysis of TMS for ASD stratified by previously defined study characteristics.

**Variables**	**Subjects (arms)**	**Proportion (95%CI)**	**I^**2**^ (%)**	***p*-value of overall effect**	***p*-value of subgroup difference**
1. TMS used as a therapeutic intervention	87 (6)	0.264 (0.181, 0.368)	0.00	<0.001	0.855
TMS used as an experimental tool	65 (5)	0.278 (0.174, 0.413)	43.67	0.002	
2. Mean age >18 years old	74 (5)	0.300 (0.204, 0.418)	26.59	0.001	0.402
Mean age ≤ 18 years old	78 (6)	0.236 (0.151, 0.350)	0.00	<0.001	
3. Target DLPFC	92 (6)	0.284 (0.200, 0.386)	0.00	<0.001	0.548
Not target DLPFC	60 (5)	0.241 (0.142, 0.379)	33.75	0.001	
4. MT% ≤ 90%	115 (8)	0.286 (0.205, 0.383)	28.77	<0.001	0.590
MT% >90%	37 (3)	0.226 (0.122, 0.380)	0.00	0.001	
5. Number of stimulation sessions <10	60(5)	0.237(0.104, 0.455)	52.64	0.002	0.680
Number of stimulation sessions ≥10	92(6)	0.293(0.207, 0.398)	0.00	<0.001	

*TMS, transcranial magnetic stimulation; ASD, autism spectrum disorder; DLPFC, dorsolateral prefrontal cortex; MT, motor threshold*.

## Discussion

This review and meta-analysis synthesized the existing data of published studies to assess the prevalence of AEs related to TMS and then further explored the potentially related factors on the AEs. We found that the overall pooled prevalence of AEs related to TMS in ASD was 25% (95% CI: 18–33%). However, given the potential bias due to the heterogeneity on sample sizes, the pathology across individuals, methodology in study design, and the low quality across the majority of the studies, the finding is not conclusive and should be considered preliminary.

We found that the AEs related to TMS on ASD were considered relatively minor. The most reported AEs were headache. The other AEs included irritability, itching, facial discomfort, sleepy, improved mood, pain at application site, and headedness/dizziness, which are consistent with the findings of Oberman et al. (3). Almost all the AEs mentioned above were mild and could be resolved after having a rest or medication. Moreover, we calculated the pooled prevalence of AEs related to TMS among ASD population by conducting a meta-analysis (overall AEs: 25%; headache: 10%; facial discomfort: 15%; irritability: 21%; pain at the application site: 6%; headedness or dizziness: 8%). These data were different from that of the previous review (35). The possible reasons for such difference are as follows: (1) many studies just reported the AEs of the overall treated patients. However, these enrolled objects are a group of patients with different diseases (i.e., attention deficit hyperactivity disorder, depression, stroke, etc.) besides ASD; (2) TMS is a relatively new technology and has not been used in patients with ASD for many years. Some studies even omitted to mention any AEs. Given the paucity of TMS safety data in ASD, the current data in published studies may be not enough to report the real prevalence of AEs; and (3) a gap in the preliminary studies of TMS in ASD is the lack of a systematic effort to identify, track, and report adverse events in study publications. As a result, it is possible that even though TMS appears to show a large safety margin, the risk of overall adverse event burden from TMS may be underestimated, especially in a vulnerable population as in individuals with ASD (22).

Besides these mild AEs mentioned above, seizure is universally known as the most serious possible TMS-related AE (22). Altogether, it is considered that the risk of seizure is <0.01% (19). However, to date, only one seizure has been reported during the treatment of TMS in ASD (29). Specifically, due to the programming error on the instrument, the subject inadvertently received a pulse of about 10 s with a frequency of 171% (RMT), which led to the seizure. This shows that the seizure event is not entirely caused by regular TMS but caused by the programming error. However, this does not mean that seizure should not be considered when TMS is used in patients with ASD. Notably, individuals who are undergoing ASD are ever boasting larger than average the prevalence of epilepsy, which is nearly 30% (36); electroencephalography (EEG) abnormalities are in the existence of about 60% of children undergoing ASD with no epilepsy (37).

With respect to the purpose of TMS, there are no significant differences in AEs prevalence between the group using TMS as a therapeutic intervention and the group using TMS as an experimental tool, even though the intensity and frequency of TMS varied. With regard to the site of stimulation, DLPFC has been pointed out as an important area in improving irritability, repetitive behaviors, and executive functioning (22). Therefore, DLPFC is one of the frequently chosen areas in TMS protocols across studies (3, 38–43). According to the results of subgroup and sensitivity analysis, no significant difference was found between the prevalence of AEs in DLPFC site and that in non-DLPFC sites. This result is different from that of a previous review (44). Maizey indicated that mild AE prevalence rates found in subsequent experimental protocols were more likely to be associated with occipital stimulation compared with other cortical sites. The possible reasons for such difference may be due to the fact that the study of Maizey et al. (44) mainly focused on the prevalence of AEs in healthy people. However, this article focuses on ASD. The included studies of this meta-analysis mainly focused on DLPFC and motor cortex. Nearly, none of the included studies stimulated the occipital cortex. Thus, different participants and lack of sufficient data may possibly lead to inconsistent results. As for MT of TMS, analyses revealed no significant association between AEs prevalence and whether the intensities used in later sessions were set above or below 90% MT. This observation is consistent with previous findings by Maizey et al. (44) and suggests that variance in MT may not be reliably predictive of AEs. With regard to the mean age of participants, there is no statistical difference between age and AEs. The risk of TMS in children appears to be similar to that in adults. This trend was also confirmed in several former studies (44, 45). However, due to the lack of sufficient data on ASD, this result needs to be interpreted with caution.

There are several strengths for this study. First, to the best of our knowledge, this is the first systematic review and meta-analysis that comprehensively summarized the pooled prevalence of AEs related to TMS. Second, when previous studies have discussed the AEs and safety of TMS stimulation, they integrated the data of several diseases. To date, there are no data specifically for the patients with ASD. The data calculated in this way may not be suitable for the future study of ASD. Our studies overcome this shortcoming and are specifically aimed at the ASD population. Third, compared with previous studies, we not only found specific AEs but also analyzed and calculated the prevalence of AEs and further explored the potentially related factors on the AEs.

Although this study has the above strengths, there are still several limitations. First, only 11 studies were included. Even though there were a number of studies focusing on the effectiveness of TMS on ASD, fewer studies reported the AEs. Second, there was heterogeneity in the methodology and low-efficacy study designs (pre-post and single-case studies). A limited number of controlled trials have been conducted over these past years. In addition, the control condition varies across studies, with the sham condition being the most utilized. Third, limited available studies were included. Thus, there is limited information for further exploration. Fourth, we did not include studies that did not report AEs. This may lead to an overestimation of the prevalence of AEs. However, we tried to contact the authors who did not report AEs to get specific data of AEs, but many authors did not reply to our email, so we could not know whether it really did not happen or was not reported. Therefore, if these studies that do not report AEs are included in our meta-analysis and the number of AE in these studies is counted as zero, it would also lead to an underestimation of the prevalence of AEs. Therefore, this study only provides preliminary data about AEs related to TMS in ASD. The results about the prevalence of AEs should be interpreted with caution. Finally, we only conducted a subgroup analysis based on four factors. Relevant factors on the prevalence of AEs associated with TMS in ASD, such as history and duration of illness variables, were not recorded in most of the papers, so their impact could not be examined. Based on the above shortcomings, it cannot be conclusively said that the results from this review can be generalized for all the ASD population.

## Conclusion

In summary, the pooled prevalence of AEs related to TMS in ASD was 25% (95% CI: 18–33%). In addition, no identified ASD-specific risk factors for TMS-induced AEs were currently found. Future studies with proper design, larger sample sizes, and stringent methodology are warranted to better evaluate the AEs of TMS in ASD.

## Data Availability Statement

The original contributions presented in the study are included in the article/Supplementary Material, further inquiries can be directed to the corresponding authors.

## Author Contributions

LK and WS-B conceived and designed the study. CB and ZD screened the literature. LY and HC conducted data collection and analysis. ZH wrote the draft of the manuscript. All authors were involved in the supply of the materials, conducted critical revision, and approved the final version for publication.

## Funding

This research was supported by Key-Area Research and Development Program of Guangdong Province (2019B030335001) and Foshan Municipal Bureau of Health (20200332). The funding agencies had no role in study design, data collection, analysis, decision to publish, or preparation of the manuscript.

## Conflict of Interest

The authors declare that the research was conducted in the absence of any commercial or financial relationships that could be construed as a potential conflict of interest.

## Publisher's Note

All claims expressed in this article are solely those of the authors and do not necessarily represent those of their affiliated organizations, or those of the publisher, the editors and the reviewers. Any product that may be evaluated in this article, or claim that may be made by its manufacturer, is not guaranteed or endorsed by the publisher.
