# Supplementary material for: Prevalence of Adverse Effects Associated With Transcranial Magnetic Stimulation for Autism Spectrum Disorder: A Systematic Review and Meta-Analysis
Source: Front Psychiatry. 2022 May 23;13:875591. doi: 10.3389/fpsyt.2022.875591 (PMC9168239; doi:10.3389/fpsyt.2022.875591)
Supplement: Supplementary file 1 [file Table_1.DOCX]

# Supplementary Table 1. Methodological Quality of included studies according to the Newcastle Ottawa Scale

| Reference | Selection | Comparability | Outcome/Exposure | Total Score |
| --- | --- | --- | --- | --- |
| Pedapati et al., 2016 | 3 | 1 | 2 | 6 |
| Ameis et al., 2020 | 3 | 2 | 3 | 8 |
| Fecteau et al., 2011 | 3 | 2 | 2 | 7 |
| Enticott et al., 2014 | 3 | 1 | 3 | 7 |
| Jannati et al., 2020 | 1 | 1 | 2 | 4 |
| Ni et al., 2017 | 2 | 1 | 1 | 4 |
| Baruth et al., 2010 | 2 | 1 | 2 | 5 |
| Yang et al., 2019 | 3 | 1 | 2 | 6 |
| Gwynette et al., 2020 | 2 | 1 | 1 | 4 |
| Oberman et al., 2014 | 2 | 1 | 1 | 4 |
| Dang et al., 2009 | 2 | 1 | 2 | 5 |
